# Microbiome symbiosis, host-pathogen dynamics, and the search for new therapeutics: highlights from the Theobald Smith Society Spring 2026 Symposium

**DOI:** 10.1128/msphere.00366-26

**Published:** 2026-08-14

**Authors:** Rachana Rao Battaje, Kyle S. Skalenko, Ahri Han, Kamilakhon Abdujabbarova, Jeffrey M. Boyd, Valerie J. Carabetta, Jason H. Yang, Srujana S. Yadavalli

**Affiliations:** 1 Center for Advanced Biotechnology and Medicine, Rutgers University 14863, Piscataway, New Jersey, USA; 2 Waksman Institute of Microbiology, Rutgers University, Piscataway, New Jersey, USA; 3 Department of Genetics, Rutgers University 198473, Piscataway, New Jersey, USA; 4 Department of Microbiology, Biochemistry and Molecular Genetics, New Jersey Medical School, Rutgers University 219171, Newark, New Jersey, USA; 5 Department of Biochemistry and Microbiology, Rutgers University 574031, New Brunswick, New Jersey, USA; 6 Department of Biomedical Sciences, Cooper Medical School of Rowan University 363994, Camden, New Jersey, USA; 7 Center for Emerging Pathogens, New Jersey Medical School, Rutgers University 67206, Newark, New Jersey, USA; Virginia-Maryland College of Veterinary Medicine, Blacksburg, Virginia, USA

**Keywords:** branch meeting, applied microbiology, environmental microbiology, mechanism discovery, microbiology, molecular biology, microbial physiology, microbiome

## Abstract

The annual Theobald Smith Society (TSS) spring meeting was convened at Rutgers University in New Brunswick, New Jersey, on 8 May 2026. TSS is the New Jersey branch of ASM and holds two annual meetings, in the fall and spring. These meetings bring together microbiologists, trainees at all levels, and professionals from both academia and industry to share their research, to network, and to engage in discussions on a wide array of topics that fall under ASM’s three main units: health, mechanism discovery, and applied and environmental microbiology. The TSS spring meeting brought together more than 140 attendees from institutions across New Jersey. This report highlights the vision and work of TSS and ASM, the breadth of research presented at the meeting through invited talks and posters, and the two keynote lectures on nutritional immunity and the modern decline of human microbiome diversity.

## INTRODUCTION

## WELCOME ADDRESS

Theobald Smith Society (TSS) president Dr. Srujana Yadavalli, assistant professor of genetics at Rutgers University in New Brunswick and principal investigator at the Waksman Institute of Microbiology, opened the meeting to welcome attendees and to highlight TSS’s various initiatives. She first introduced the national ASM organization and encouraged the attendees to become ASM members if they were not already. ASM provides platforms for members to connect with peers as well as scientists and experts through field-specific programs, webinars, and meetings such as TSS. Members can also develop their career paths through academic- and industry-focused informational panels and professional development programming. Dr. Yadavalli also highlighted opportunities to engage in leadership roles within ASM at both the local and national levels. She concluded with a reminder for all who are interested to register for the ASM Microbe national conference held in Washington, DC, from 4 to 7 June 2026.

Dr. Yadavalli continued her welcome address with a brief introduction to TSS and the pioneering scientist after whom the society is named: Dr. Theobald Smith. Dr. Smith was a pathologist and bacteriologist famously known for his extensive work on Texas cattle fever. In addition to his involvement and contributions to the fields of microbial pathogenesis and immunology, he is also remembered as a renowned physician, teacher, and inventor. Continuing this spirit and enthusiasm for science and education, TSS is New Jersey’s regional ASM branch that aims to build a network of scientists across all New Jersey institutions in order to exchange data and ideas openly and freely and to promote scientific engagement. Those who would like to learn more about TSS can find information on TSS’s website (https://www.njmicrobe.org/join).

Dr. Yadavalli also introduced the 2025–2026 TSS officers: herself as president; Dr. Jason Yang from Rutgers New Jersey Medical School as president-elect; Dr. Valerie Carabetta from Cooper Medical School of Rowan University, Camden, as treasurer; Dr. Jeffrey M. Boyd from Rutgers University–New Brunswick as branch counselor; and Dr. Raymond Sullivan from Rutgers University–New Brunswick as secretary. Additionally, she took a moment to recognize Dr. Sullivan for his dedicated involvement with the Theobald Smith Society and his years of service as secretary as he stepped down from the position. His contributions were acknowledged as an important part of TSS’s continued mission to promote microbiology research, foster scientific exchange, and strengthen community engagement in New Jersey. She also announced that the positions for the next president-elect and secretary are open and encouraged those interested to apply. Moreover, the welcome address also congratulated the three recipients of the Peggy Cotter Award, which provides funds for early-career scientists who are members of TSS to attend ASM Microbe: Dr. Nadine Alvarez, supervisor and research assistant at Hackensack Meridian Health; Dr. Rohan Maddamsetti, assistant professor at Rutgers University–New Brunswick; and Dr. Shivangi, postdoctoral associate at Rutgers University–Newark.

Finally, Dr. Yadavalli concluded her opening remarks by briefly highlighting the fall 2025 TSS meeting held at Cooper Medical School of Rowan University in Camden, NJ, extending thanks to the keynote lecturers, oral presenters, and poster winners as well as reviewing the spring 2026 TSS meeting agenda.

The opening remarks were followed by a brief presentation titled “Perks of Joining ASM” led by Dr. Liisa Veerus, ASM US Young Ambassador for the greater New Jersey area, and an “Overview of ASM Journals” by Joseph Schwartz, assistant managing editor for ASM’s *Microbiology Spectrum* journal.

## KEYNOTE ADDRESS

The first keynote address was delivered by Dr. Jeffrey Boyd, a professor of biochemistry and microbiology and the director of the Microbial Biology Graduate Program at Rutgers University–New Brunswick. Additionally, he has been a part of TSS leadership, serving as president (2018–2020) and as ASM branch councilor (2022–present). Dr. Boyd’s research examines how *Staphylococcus aureus* senses and responds to stress and the impact of this stress response on physiology and infection outcomes.

In his address, Dr. Boyd focused on the genetic regulation of iron ion homeostasis and highlighted ongoing research from his laboratory, primarily on the recent work of Liangyu Lyu, a current second-year PhD student. Later in his lecture, he briefly discussed the work conducted by Kylie Ryan Kaler and Karla Esquilin-Lebron, the previous two postdoctoral fellows in his lab, on a protein known as Fpa (1).

Dr. Boyd studies community-acquired MRSA, which asymptomatically colonizes ~2% of the population yet causes severe infections, including sepsis, endocarditis, and necrotizing pneumonia, and ranks among the leading causes of infection-associated mortality in the United States and worldwide. He emphasized to his audience that MRSA and broader antimicrobial resistance represent an evolving, urgent public health challenge requiring sustained attention from scientific and medical communities.

Dr. Boyd and his team focus specifically on identifying novel bacterial targets and expanding the repertoire of cellular targets, antimicrobial agents, and therapeutic strategies to treat resistant *S. aureus* infections. Specifically, their goal is to identify and describe novel targets that a structural biologist or biochemist can later design a compound to inhibit or inactivate. In today’s address, Dr. Boyd focused on iron (Fe) ion homeostasis in *S. aureus*. Iron homeostasis is a vital process in this organism because *S. aureus* must uptake, properly traffic, and utilize ions to support critical cellular functions, including the synthesis of essential iron-sulfur (Fe-S) cofactors, which play key roles in metabolism and regulation (2, 3). Importantly, disruption of Fe acquisition and homeostasis, or of Fe-S cluster biogenesis, can impair bacterial growth and negatively impact virulence (4). Dr. Boyd highlighted that many components of these bacterial pathways differ significantly from those found in eukaryotic organisms, making them promising targets for the development of novel antimicrobial therapies.

Dr. Boyd noted that during infection, the bacterium is engaged in a continual battle with the host for access to essential metal ions. In response, the host employs a process called nutritional immunity, in which it sequesters Fe to limit bacterial growth (5). In response, *S. aureus* utilizes eight described Fe uptake systems, highlighting the importance of Fe acquisition for the organism’s survival and pathogenicity. Furthermore, *S. aureus* produces two siderophores, can use siderophores produced by other microorganisms, produces a broad-spectrum metallophore, and can acquire Fe using a heme uptake system. Given that the *S. aureus* genome is about 3.5 megabases, a substantial portion of it is dedicated to Fe uptake, transport, and utilization. Collectively, these mechanisms highlight the central role of Fe in the physiology and virulence of *S. aureus* (6).

On the other hand, excessive Fe uptake and cytosolic accumulation can become toxic to *S. aureus*; therefore, the organism must tightly regulate iron homeostasis (7). Dr. Boyd highlighted two proteins involved in preserving this fine balance: the ferric uptake regulator (Fur) and FpA. Fur protein is a transcription factor that functions as a repressor of Fe acquisition genes (Fur operon) when intracellular Fe levels are sufficient. Fpa binds Fe when Fe levels are sufficient, promoting Fur repression. However, under Fe-depleted conditions, Fpa becomes demetallated and subsequently forms a complex with Fur, alleviating Fur’s transcriptional repression and leading to transcription of the Fur regulon (1). Dr. Boyd presented unpublished results elucidating the molecular mechanisms underlying Fur-Fpa interactions, including the specific residues involved in protein-protein interaction and iron sensing. He also highlighted functional differences between Fur from gram-negative and gram-positive bacteria, providing interesting insights into how pathogenic bacteria maintain tightly regulated Fe homeostasis during infection. These findings may contribute to the development of novel antimicrobial therapeutics that can strengthen the existing arsenal for combating emerging infections caused by *S. aureus* strains, including MRSA and multidrug-resistant isolates.

In closing remarks, Dr. Boyd highlighted and acknowledged Dr. Ray Sullivan’s valuable contributions as TSS secretary over the past 5 years. He mentioned that Dr. Sullivan earned his MS and PhD from Rutgers, New Brunswick, and then pursued a career as a scientist at the U.S. Department of Defense. Dr. Sullivan has been a visiting scientist at Rutgers since 2019. He constructed and maintained the TSS website and social media presence, contributing directly to TSS’s growth and to scientific dissemination within the community.

## 2026 YOUNG INVESTIGATOR AWARD

At the meeting following the keynote lecture by Dr. Boyd, president-elect Dr. Yang presented the TSS Young Investigator Award to Dr. Cameron Myhrvold from Princeton University. Dr. Myhrvold is an assistant professor in the Department of Molecular Biology, where he runs his own research group studying transcription and RNA biology in the context of viruses and viral detection using CRISPR-based technologies.

## WAKSMAN AWARD LECTURE

Dr. Valerie J. Carabetta of Rowan University presented the 2026 Waksman Award & Lectureship to Dr. Martin J. Blaser of the Rutgers Robert Wood Johnson Medical School, who serves as the Henry Rutgers Chair of the Human Microbiome, director of the Center for Advanced Biotechnology and Medicine (CABM), and professor of medicine and pathology and laboratory medicine at Rutgers University. Dr. Blaser has devoted his career to exploring the long-standing relationships of colonizing bacteria with human hosts. For three decades, his research centered on *Campylobacter* species and *Helicobacter pylori*, organisms that have now proven invaluable as model systems for studying how resident bacteria interact with their hosts. Over the past two decades, his focus has broadened to encompass the role of the human microbiome in a wide range of significant health conditions, including asthma and allergy, obesity, diabetes, neurological disorders, and cancer. He has 28 U.S. patents and has authored more than 600 original research articles. His book, *Missing Microbes*, has been translated into 20 languages.

Dr. Blaser began with a poignant tribute to three pioneering figures in microbiology: Dr. Theobald Smith, who identified tick-borne disease transmission in Texas cattle fever; Dr. Selman A. Waksman, the Nobel laureate from Rutgers University who birthed the antibiotic era through his discovery of streptomycin; and Dr. Sergei N. Winogradsky, a foundational figure in soil and environmental microbiology. Dr. Blaser shared a serendipitous moment of historical continuity: just two days prior to this talk, he was gifted a copy of the rare book, 1953’s *Sergei N. Winogradsky—His Life and Work—The Story of a Great Bacteriologist*, authored by Selman A. Waksman himself.

In his compelling lecture, titled “Missing in Action: Characteristics and Consequences of an Invisible Extinction,” Dr. Blaser outlined a silent modern crisis: the generation-spanning depletion of the human microbiota (8). The “invisible extinction” refers to a global shrinkage of gut microbial diversity that directly tracks with the socioeconomic advancement of human populations (9). For millennia, human health relied on the uninterrupted vertical transmission of maternal microbes to offspring. Today, modern practices, ranging from widespread antiseptic exposure and surging Cesarean section rates to aggressive early-life antibiotic use, have severed this chain. The resulting depletion of our collective microbiome is a baseline shift that Dr. Blaser and other scientists are now linking directly to several chronic modern pathologies (10).

The primary clinical focus of the lecture was the growing asthma epidemic, particularly among children and young adults. Dr. Blaser raised the central question: “Is an altered microbiota an important risk factor for the asthma epidemic?” To systematically answer this, Dr. Blaser outlined a framework combining four distinct lines of evidence: theoretical considerations regarding modern birthing and sanitization practices, clinical investigations tracking real-world human infant cohorts, epidemiological studies establishing vast population-level correlations, and experimental evidence proving biological causality *in vivo* through mouse models.

For decades, farmers have fed subtherapeutic doses of antibiotics to livestock to accelerate growth, prompting Dr. Blaser to investigate whether antibiotics induce a similar developmental and metabolic phenomenon in mammals. Using mouse models, Dr. Blaser’s lab demonstrated that combining early-life antibiotic exposure with a high-fat diet creates a powerful synergy that permanently accelerates body fat accumulation. While the initial disruption to the gut microbiome is often temporary, the resulting metabolic and immune alterations last much longer in the host. Several parallel clinical- and population-level studies established the connection between early-life exposure to antibiotics and common childhood-onset health conditions, including asthma and allergic rhinitis. Patrick et al. (11) provided population-level proof by demonstrating that a community-wide reduction in pediatric antibiotic prescribing practices directly correlated with a measurable, population-wide decrease in childhood asthma incidence (11). COPSAC_2010_ birth cohort study of 623 Danish children demonstrated that the heightened asthma risk seen in Cesareansection births correlated directly with abnormal microbial maturation scores (12).

To explain the cellular mechanics behind this phenomenon, Dr. Blaser highlighted a recent breakthrough showing how early-life antibiotic exposure disrupts the normal development of the gut microbiota and its impact on the neonatal immune system. Using a perinatal ampicillin exposure model, Dr. Blaser and colleagues demonstrated that the dysbiotic microbiota impairs immune functions by disrupting dendritic cell function and preventing the generation of regulatory T cells needed to control inflammation (13). Without these cells, the body cannot modulate its immune response, leading to severe airway pathology. *In vivo* studies show this immune failure translates directly into increased airway resistance, decreased lung elasticity, and pronounced wheezing, alongside permanent structural scarring of Peyer’s patches in the gut (14, 15). Validating these findings, Dr. Blaser’s lab used network modeling to prove that transplanting immature human infant microbiotas into germ-free mice perfectly replicates these exact systemic immune deficiencies in the next generation.

Looking forward, Dr. Blaser concluded with an optimistic call to action, emphasizing that the invisible extinction is a solvable problem. He advocated for a two-pillar strategy: minimizing toxic, unnecessary antibiotic exposures to the early-life microbiome and developing targeted preventative and restorative regimens (8). However, he left the audience with an unanswered clinical question: “If we are going to restore an infant’s microbiome, should we utilize the modern mother’s microbiome, which may already be depleted, or look toward baby unaltered microbiomes as the standard?” Ultimately, Dr. Blaser’s lecture underscored that our early-life microbial partnerships are fundamental determinants of lifelong health, urging that science must prioritize restoring the ancestral microbial partnerships disrupted by modern medicine and lifestyle before their loss becomes irreversible.

## PRESENTATION THEMES

ASM comprises three thematic units that span diverse microbial fields that the organization aims to promote and advance: ASM health, mechanism discovery, and applied and environmental microbiology. Similar to recent meetings, the spring 2026 meeting had oral presentations and posters that were well balanced under these units. The six oral presentations were evenly divided among these units, and the posters had a similar distribution: 14 out of 66 posters fell under ASM health (21.2%), 25 fell under ASM mechanism discovery (37.9%), and 27 fell under ASM applied and environmental microbiology (40.9%). The keynote lecture by Dr. Boyd shared mechanistic insights into nutritional immunity at the host-*S. aureus* interface, while the Waksman Award lecture by Dr. Blaser highlighted the invisible extinction of microbes affecting the health of modern life, including alarming asthma trends. Taken together, all three ASM units were well represented at the spring meeting and fostered insightful discussions and engaging Q&A sessions.

### ASM Health

Representing ~21% of the science presented at this event, the ASM Health category centered heavily on translational disease interventions, host-pathogen interactions, and next-generation pharmacological designs. Some of the major themes focused on drug screening; pathogenesis and virulence; and clinical diagnostic and detection tools.

In the drug screening category, Carissa Colfer (Montclair State University, Montclair, NJ) presented her work on “Selective Competitive Inhibition of *Wuchereria bancrofti* (Wb) Dihydrofolate Reductase by a Novel Antifolate.” Wb is a parasitic worm and is the causative agent for elephantiasis, which affects 50 million people worldwide. Her work focuses on identifying selective inhibitors against the Wb dihydrofolate reductase. Initially, an *in silico* screen was performed to identify drugs that bound homologs of dihydrofolate reductase. Their screen identified 194 potential drug targets that bound dihydrofolate reductase. Carissa interrogated one of the hits from her screen (TSD13) and found that TSD13 moderately inhibited the Wb dihydrofolate reductase with an IC50 of ~16 µM. TSD13 was also selective toward Wb dihydrofolate reductase, where TSD13 bound human dihydrofolate reductase with an IC50 of ~340 µM. Their group obtained the crystal structure of Wb dihydrofolate reductase complexed with TSD13 and identified major residues that interact with TSD13. Collectively, their data identify a potential selective lead compound that targets Wb dihydrofolate reductase.

In the pathogenesis and virulence category, Joycelyn Radeny (Rutgers New Jersey Medical School, Newark, NJ) presented her work on “*Mycobacterium tuberculosis* Effector Ppe61 Targets Macrophage Mitochondria.” Mtb is an intracellular bacterium and is the leading cause of global deaths by an infectious agent. Her work focuses on identifying poorly characterized members of the PE/PPE protein family that target host mitochondria to promote bacterial survival. Initially, an *in silico* screen using mitochondrial targeting sequence prediction software was performed to evaluate all 170 members of the family. Their screen identified several PE/PPEs that might localize to the mitochondria, including the uncharacterized effector PPE61, whose localization was confirmed using immunofluorescence. She further interrogated changes to the mitochondria induced by this effector. She found that PPE61 induced fragmentation of mitochondrial networks, increased expression of critical stress response genes (Sod1, Sod2, Hspd1, and Hspe1), and induced mitophagy. Their group conducted Seahorse extracellular flux assays and observed that PPE61 significantly reduced basal mitochondrial respiration and ATP production, though surprisingly without lowering mitochondrial fitness or membrane potential. Collectively, their data present a novel mechanism through which an Mtb virulence strategy modifies host cell dynamics to alter oxidative phosphorylation, which can be targeted by future host-directed therapies.

Lastly, in the clinical detection tools category, Sadaf Fatima (Montclair State University, Montclair, NJ) presented her work on “Development of Rapid Field-Testing Kit for Detection of Ranavirus and Other Viral Aquatic Pathogens.” Ranavirus is a viral pathogen and is the major contributor to the sharp global decline in amphibian and aquatic populations since the early 20th century. Her work focuses on addressing current testing methods that are expensive, time-consuming, and impractical for fieldwork. Initially, a novel aptamer discovery technique called dual-tag systematic evolution of ligands by exponential enrichment was performed to generate short single-strand DNA sequences. Their screen identified aptamers that target the virus’s major capsid protein (MCP). She further interrogated the binding affinity and specificity between these aptamers and the viral target. She found that employing scrambled aptamers as negative controls allowed her group to analyze and validate selectivity by comparing distinct binding patterns. Their group aims to transition this platform into a lateral flow format for on-site diagnostic applications. Collectively, their data present a potential rapid field test that can help in the on-site detection of ranavirus.

By combining the structural discovery of new targeted drugs with fast, on-site testing kits, these projects lay a strong groundwork for more accessible and effective medical treatments.

### ASM Mechanism Discovery

The meeting showcased ~38% of the presentations (2 oral and 25 posters) that were under the ASM Mechanism Discovery unit. These works covered a wide range of topics, such as bacterial fitness during stress, enzyme dynamics, protease function in nitric oxide response, bacterial morphology, bioenergetic stress and antibiotic persistence, and phage biology during quorum sensing. While bacteria were the most well represented under this unit, the poster session had one virus-related poster, two fungus-related posters, and one dinoflagellate-related poster that fell under the mechanism discovery category.

Yuxuan Wang, a PhD student from Rutgers Health-Robert Wood Johnson Medical School, gave an invited talk on her work studying the mechanisms of the transcriptional regulation of a gene expression repressor called HrcA in *Chlamydia* species. *Chlamydia* is one of the most common sexually transmitted pathogens that often goes undetected, and treatment for this infection still relies on broad-spectrum antibiotics. As an obligate intracellular pathogen, *Chlamydia* requires optimal conditions in order to complete its developmental cycle, accumulate within the host cell, and eventually be released. Additionally, *Chlamydia* exhibits persistence: a bacterium’s ability to survive antibiotics in a dormant state under extreme stress, such as antibiotic treatments, nutrient starvation, and heat shock. Because HrcA is an important protein for the bacteria to survive and adapt to heat shock, her work aimed to analyze the HrcA regulon. By performing chromatin immunoprecipitation-quantitative PCR, she identified a highly conserved gene, *ctl0271*, that interacted with HrcA and observed that the expression of this gene decreased when HrcA was overexpressed. This gene was subsequently named *hagF* (for heat-activated HrcA-repressed gene F), and the protein it encodes was HagF. To begin determining the function of this protein, she created knockdown strains of *hagF* using CRISPR silencing and found that this gene is most likely not essential since bacteria lacking HagF did not affect the bacteria’s developmental cycle under optimal conditions. Only at high temperatures was a defect in the developmental cycle observed. She proposed a possible model for HagF regulation of HrcA function in that HagF may be preventing HrcA binding to its transcriptional element to relieve HrcA repression of downstream genes (negative feedback loop), especially since silencing *hagF* resulted in the reduced expression of downstream heat shock genes. She hopes to continue her work by next utilizing AlphaFold-based structural prediction approaches to determine how HagF might be inhibiting HrcA dimerization required for gene expression repression as well as clarify the model of HrcA regulation based on these data.

Alexandra Kwasnik from Rutgers University–New Brunswick gave a poster presentation of her work investigating bacterial response to host-derived nitrogen oxide (NO) in *Pseudomonas aeruginosa*. She is currently a master’s student at the School of Environmental and Biological Sciences and won one of the meeting’s three best poster prizes at the meeting. Host-derived NO disrupts bacterial proteome homeostasis. Through transcriptional profiling, she observed differential expression of bacterial genes in a mutant for the Lon protease versus wild-type bacteria under NO stress. The Lon protease is important for proteome homeostasis in bacteria because it targets specific unfolded/inactivated proteins for degradation. Alexandra observed differential regulation of different iron-sulfur cluster proteins and looked deeper into potential Lon targets. She hypothesized that one protein that Lon may be targeting for degradation during NO stress is the enzyme aconitase, an iron-sulfur enzyme important for iron homeostasis. Hence, she hypothesized that Lon can target and inactivate aconitase, leading to its degradation. She measured aconitase activity in Lon mutant bacteria versus wild-type bacteria and found a decrease in aconitase activity in the Lon mutant strain. She also observed that a loss of Lon reduced bacterial growth during NO stress while growing in TCA carbon sources (which were used because aconitase is an important member of the TCA cycle). With these observations, she aims to next perform Western blots of tagged aconitase to determine whether Lon is able to selectively target and degrade aconitase.

Stephen Anyanwu, a master’s student from Rutgers Health-New Jersey Medical School, presented a poster on his project investigating how disruption in metabolism affects antibiotic survival and pathogenicity of pathogenic *Escherichia coli*. Antimicrobial resistance and persistence continue to remain a significant public health issue globally. Previous work has shown that antibiotic-induced depletion of ATP can facilitate persistence, but whether ATP levels directly impact bacterial survival during antibiotic-induced stress is not clearly understood. He found that changes in cellular energy levels, like ATP, can promote antibiotic tolerance and persistence in laboratory strains. He extended the study to enterohemorrhagic and enteropathogenic *E. coli* strains and observed that changes in energy levels can also influence virulence. His project also involves the development and validation of a dual expression-optogenetic system in *E. coli* that can tightly control the induction of cellular ATP levels in order to study bioenergetic stress independent of antibiotics. This work suggests that energy homeostasis is a key regulator of bacterial survival and pathogenicity and aims to further explore this in order to identify targets for treating antibiotic-resistant infections.

Francis Santoriello, a postdoctoral fellow at Princeton University, presented a poster on his research into a family of phages that detect quorum sensing. His work focuses on understanding phage transmission to new hosts and the cues that induce lysis under high cell density. Bacterial density is tightly regulated by quorum sensing, a type of cell-cell communication that is associated with the release and detection of extracellular molecular signals. His phage of interest, called ϕVP882, encodes a protein, VqmAϕ, that is a homolog of a quorum-sensing receptor found in *Vibrio* called VqmA. VqmAϕ enables the phage to detect the autoinducer protein DPO (3,5-dimethylpyrazin-2-ol). Through his project, Francis hypothesized that phages can sense DPO, which is abundant in nature due to the highly conserved characteristics of DPO in bacteria. To determine this, he queried databases for sequences of linear plasmid phages and identified phages similar to VP882 in multiple species of bacteria found in clinical and environmental isolates. He found DPO-dependent VqmAϕ proteins can enable VP882-like phages carried by DPO-producing bacteria to detect and respond to bacterial cell density. Francis was one of the three poster prize winners at this meeting.

### ASM Applied and Environmental Microbiology

Making up ~41% of the symposium’s presentations, the ASM Applied and Environmental Microbiology track spotlighted how microbial ecosystems can be engineered, profiled, or decoded to solve modern environmental and agricultural challenges. Some of the major themes focused on environmental bioremediation and contaminant degradation, symbiotic relationships, and the development of environmental surveillance technologies that include field devices and data analysis pipelines for long-read Nanopore rRNA sequencing and ecological labeling using large language models (LLMs).

In the bioremediation and contaminant degradation section, Matthew P. Finegan (Rutgers University–New Brunswick, New Jersey) presented his work, titled “Impacts of Antibiotics on Freshwater Plastisphere Communities.” His work focuses on an important topic regarding environmental contamination with microplastics and the bacteria that grow on them, called the plastisphere. Microplastics serve both as a surface for bacterial communities to form, leading to a reservoir for antibiotic resistance genes, and as a sponge for organic contaminants, such as pharmaceuticals (including antibiotics), from improperly treated wastewater, exposing bacterial communities to subinhibitory concentrations of antibiotics. The researchers wanted to shed light on how exposure to antibiotics influences the structure and function of the plastisphere. To show this, riverine microcosms were mixed with tiny pellets of common plastics found in the environment, such as high-density polyethylene, polystyrene, and polyethylene terephthalate, for a week, and then these plastisphere communities were challenged with an antibiotic mixture. Using the Oxford Nanopore Technologies MinION to sequence the rRNA of the bacterial species present in the biofilm, their results showed that the composition of the biofilm growing on the plastic differed from the planktonic cell population. The group aims to further understand how the composition of the biofilm changes and determine the prevalence of antibiotic resistance genes within the biofilm.

Junwon Yang, a postdoctoral research associate at the New Jersey Institute of Technology, presented a poster on his work investigating biostimulation strategies to remove dioxanes, an emerging contamination problem. Junwon was also a winner of the poster prize at the meeting. His research focuses on a group of enzymes called propane monooxygenases (PRMs) that catalyze the oxidation of 1,4-dioxane, a trace contaminant found in the environment and in many goods. Because propane, an inexpensive and readily available compound, can induce PRM expression, Junwon was interested in testing the catalytic abilities of PRMs from bacteria, specifically mycobacteria, as a novel propane-based approach to dioxane removal. From his work, he identified substrate affinities and catalytic capacities of PRMs in *Mycobacteria*, specifically *M. dioxanotrophicus*, *M. marinum*, and a *Mycobacteria* species strain, ENV421, which is known to metabolize a variety of chemicals when grown solely on propane. He also performed a structural analysis on his PRMs of interest and found that differences in catalytic ability may depend on structural differences, such as the hydrophobicity of the active sites. With his data, he hopes to leverage the enzyme kinetics of microbial populations to apply this strategy toward decontaminating dioxane-polluted environments.

In the symbiotic relationship section, Ian M. Oiler (Rutgers University–Newark, New Jersey) presented his work titled “Genomic Insights into Bacterial Isolates Dominating Honeypot Ant Crop Microbiomes Reveal Metabolically Distinct *Fructilactobacillus* sp.” His work focused on further understanding the repletism phenotype, where a honeypot ant stores large amounts of food in its digestive tract and supports a colony by providing the stored food, and the microbiome associated with the gut. His work expands on isolating and characterizing previously identified bacteria within the gut of the honeypot ant. He showed that these bacteria represent two evolutionarily distinct lineages, but both were most closely related to *F. fructivorans*. Collectively, his data provide a roadmap to understanding the mechanism of repletism in ants.

Lastly, in the technologies section, many groups presented novel technologies to monitor bacteria within the environment or unique computational pipelines; however, the poster that stood out was “Zero-Shot Ecological Annotation of Microbial Genomes with myLLannotator Accelerates Scientific Discovery” by Alyssa Lu Lee (Rutgers University–New Brunswick, New Jersey). Her work outlines the development of a computational tool called myLLannotator, using LLMs. The tool is a Python package that can annotate about 18,000+ genomes in about 2 hours on a conventional laptop. Using their tool, the group showed that duplicated genes are depleted in endosymbiotic bacteria. By pairing natural field observations with fast computing and sequencing tools, these diverse studies pave the way for cleaner ecosystems and smarter environmental tracking.

## CONCLUSION

The Spring 2026 Symposium of the Theobald Smith Society showcased the breadth and depth of cutting-edge microbiology research, reflecting the remarkable diversity of questions being pursued by New Jersey’s microbiology community. Topics ranged from molecular mechanisms underlying bacterial iron homeostasis, stress responses, and antibiotic resistance, to environmental investigations spanning deep-sea hydrothermal vents, freshwater ecosystems, and agricultural settings. Linking fundamental microbial physiology to urgent public health challenges, the meeting underscored that addressing the most pressing issues in microbiology will require continued collaboration across disciplines, career stages, and institutions.
